# A CART-based prognostic model for risk stratification of postoperative early recurrence in hepatocellular carcinoma with microvascular invasion

**DOI:** 10.3389/fonc.2025.1655739

**Published:** 2025-10-24

**Authors:** Jie Zeng, Ri-Jin Lu, Zheng Tao, Can Zeng, Kai-Xiang Mo, Weijie Cen, Yan Lin, Rong Liang, Le-Qun Li, Guo-Bin Wu, Jia-Zhou Ye, Rong-Yun Mai

**Affiliations:** ^1^ Department of Hepatobiliary & Pancreatic Surgery, Guangxi Medical University Cancer Hospital, Nanning, China; ^2^ Department of Physiology, School of Basic Medical Sciences, Guangxi Medical University, Nanning, Guangxi, China; ^3^ Guangxi Liver Cancer Diagnosis and Treatment Engineering and Technology Research Center, Nanning, China; ^4^ Department of Digestive Oncology, Guangxi Medical University Cancer Hospital, Nanning, China

**Keywords:** hepatocellular carcinoma, hepatectomy, microvascular invasion, classification and regression tree, early recurrence, recurrence-free survival, overall survival

## Abstract

**Background:**

Postoperative early recurrence (ER) poses a major threat to long-term survival in hepatocellular carcinoma (HCC), especially in patients with microvascular invasion (MVI). Although conventional staging systems provide prognostic guidance, they are often inadequate for capturing recurrence risk in this high-risk subgroup. To develop and validate a CART-based prognostic model tailored to ER risk stratification and assessment of long-term outcomes following curative hepatectomy in MVI-positive HCC.

**Methods:**

A retrospective cohort of 440 patients with histologically confirmed HCC and MVI who underwent curative resection was analyzed. ER-associated predictors were identified via multivariable Cox regression and used to construct a classification and regression tree (CART) algorithm. Model discrimination, calibration, and clinical utility were evaluated using time-dependent ROC curves and decision curve analysis. Predictive performance for recurrence-free survival (RFS) and overall survival (OS) was compared against established staging systems.

**Results:**

Eight independent factors predictive of ER were identified: HBV-DNA load, tumor size, Edmondson-Steiner grade, tumor capsule integrity, MVI classification, satellite nodules, Ki-67 index, and CK19 expression. The CART model demonstrated robust discriminative ability (C-statistic: 0.773 in training; 0.764 in validation), and consistently outperformed conventional staging systems. Furthermore, CART-defined risk strata were significantly associated with both RFS and OS (*P* < 0.001).

**Conclusions:**

This CART-based framework provides a transparent and clinically implementable tool for ER risk stratification in MVI-positive HCC. By outperforming existing staging algorithms, it offers a basis for individualized surveillance and postoperative management.

## Introduction

Hepatocellular carcinoma (HCC) is one of the most prevalent and lethal malignancies worldwide, accounting for a major share of cancer-related mortality (1–3). Although advances in surgical techniques and perioperative management have improved procedural safety, long-term outcomes following curative liver resection remain unsatisfactory. Up to 70% of patients experience tumor recurrence within five years after surgery (4), underscoring the need for more accurate and individualized risk stratification strategies—particularly for predicting early postoperative relapse, where robust prognostic tools are still lacking.

Among histopathological features, microvascular invasion (MVI) has emerged as one of the strongest and most consistent predictors of early recurrence and reduced survival. MVI is histologically defined as the presence of tumor emboli within endothelial-lined vascular spaces in the peritumoral liver parenchyma. Its presence reflects an aggressive tumor phenotype characterized by intrahepatic micrometastasis and vascular dissemination (5). However, MVI cannot be reliably detected preoperatively via current imaging modalities and is typically confirmed only through postoperative pathological examination (6). Consequently, despite its high prognostic value, MVI remains underrepresented in conventional staging systems—highlighting the urgent need to integrate biologically driven markers into recurrence prediction models (7, 8).Even among patients undergoing liver transplantation or early-stage HCC, the presence of MVI is associated with poorer outcomes (9).

Postoperative recurrence in HCC is commonly categorized into two biologically distinct subtypes based on timing and clonal origin: early recurrence (ER), defined as relapse within two years post-surgery, and late recurrence (LR), occurring thereafter. ER is believed to result from intrahepatic micrometastases disseminated before or during surgery, representing a monoclonal expansion of the primary tumor (10, 11). In contrast, LR typically arises via polyclonal *de novo* tumorigenesis against a background of cirrhosis or chronic inflammation (10). Clinically, ER is associated with more aggressive tumor behavior, limited salvage options, and substantially poorer survival (12). Its temporal proximity to surgery renders ER a clinically actionable window, wherein timely and accurate risk identification may enable early intervention and tailored postoperative surveillance. ER rates in MVI-positive HCC patients have been reported to exceed 76.7%, with a median recurrence-free survival less than 12 months (13). Despite this, no universally accepted risk classification system currently exists for ER in this population (13).

Although widely used in clinical practice, staging systems such as the Barcelona Clinic Liver Cancer (BCLC), tumor-node-metastasis (TNM), China Liver Cancer (CNLC) and other classifications were originally designed to guide treatment allocation and predict overall survival (14–20). However, their ability to stratify ER risk—particularly in patients with MVI—remains limited. These systems are typically built on linear assumptions and fixed variable hierarchies, which inadequately capture the nonlinear and multifactorial biological processes that drive early relapse. Their suboptimal performance in this context has been corroborated by multiple retrospective validations. In response to these limitations, machine learning–based tools have emerged as promising alternatives for individualized risk prediction in oncology. Among these, classification and regression tree (CART) models offer interpretable, rule-based decision frameworks capable of capturing complex interactions and hierarchical variable importance through recursive partitioning (21, 22). Unlike black-box algorithms, CART is transparent and highly adaptable to clinical settings, making it particularly suitable for bedside risk stratification in heterogeneous HCC populations.

In this study, we developed and validated a CART-based prognostic model to stratify early recurrence risk in HCC patients with histologically confirmed MVI following curative hepatectomy. We further compared its predictive performance against conventional staging systems and evaluated its association with recurrence-free survival (RFS) and overall survival (OS). By identifying patients at elevated risk of early relapse, the model aims to inform risk-adapted surveillance protocols, guide adjuvant therapy planning, and ultimately improve outcomes in this biologically aggressive subset of HCC.

## Methods

### Patient selection

This retrospective cohort study included patients with hepatocellular carcinoma (HCC) who underwent curative-intent hepatic resection at Guangxi Medical University Cancer Hospital between September 2013 and June 2019. Patients were eligible if they met the following criteria: i) good preoperative hepatic function background; ii) initial hepatectomy with therapeutic intent; and iii) postoperative pathological diagnosis confirmed as HCC complicated with MVI. The exclusion criteria were: i) preoperative received any antitumor therapy; ii) preoperative tumor infiltration of portal veins, hepatic veins or adjacent organs; iii) other malignancies simultaneously; iv) postoperative hospital death; and v) incomplete clinical data. Ultimately, a total of 440 eligible patients were identified. The cohort was randomly split into a training set (n = 329) and a validation set (n = 111) using a 3:1 allocation ratio (**Supplementary Figure 1**). The study protocol was approved by the Institutional Review Board of Guangxi Medical University Cancer Hospital (**Approval Number: KY2025644**) and complied with the Declaration of Helsinki.

### Definition and classification of microvascular invasion

MVI was defined as the presence of tumor emboli within vascular spaces lined by endothelial cells, as confirmed by microscopic histopathology (6). The classification system for MVI was based on prior studies and defined as follows: M0, indicating no microvascular invasion; M1 (low-risk), defined as ≤5 microvascular invasion sites located within 1 cm of the tumor margin; and M2 (high-risk), defined as >5 sites or any site located more than 1 cm from the tumor margin. The number of MVI foci was determined by reviewing all available histological sections, and the distance was measured as the shortest path from the tumor edge to the nearest MVI focus (6).

### Staging system classification

All included cases were staged by: China Liver Cancer (CNLC) staging (14); 7th edition of TNM/AJCC (TNM) staging (15); Barcelona Clinic Liver Cancer (BCLC) staging system (16); French staging (17); Okuda staging (18); Japan Integrated Staging (JIS) (19); and Cancer of the Liver Italian Program (CLIP) staging (20).

### Hepatectomy and follow-up

Hepatectomy was carried out based on preoperative imaging that showed complete resectability of all tumors within the liver’s functional capacity. Further information and criteria for liver resection can be found in our previous research.

Patients were followed up using a similar approach as outlined in our previous study (11). In cases of recurrence during the follow-up period, patients were treated with optimal therapeutic methods, including hepatic artery infusion chemotherapy, transcatheter arterial chemoembolization, systemic chemotherapy, targeted therapy, radiofrequency ablation, and secondary hepatectomy. ER was defined as recurrence within 2 years following hepatectomy (11, 13). RFS was measured from the date of the surgery to the date of tumor recurrence. OS was defined as the time between operation and death (23).

### Establishment of a CART strategy

To identify independent prognostic variables associated with early recurrence (ER), a two-step analytical approach was adopted. First, univariate Cox regression analysis was performed to evaluate the association between each candidate variable and ER. Variables with a significance level of P < 0.05 were considered statistically significant and were subsequently included in the multivariate analysis. In the second step, multivariate Cox regression analysis was conducted using these significant variables to identify independent prognostic factors for ER.

Subsequently, a CART model was developed based on the results derived from the multivariate Cox regression analysis. CART is a machine learning technique that uses recursive partitioning to create a decision tree for predicting categorical or continuous outcomes. It works by repeatedly splitting the data into subsets based on predictor variables that maximize homogeneity in the outcome within each resulting subgroup (24, 25). This approach allows the identification of specific combinations of prognostic indicators that are most predictive of ER.

The CART model was implemented using the Classification Decision Tree module in SPSS version 26.0 (IBM Corp., Armonk, NY). This method facilitates the development of a structured, easy-to-interpret decision tree that captures nonlinear relationships and interaction effects among predictors, thereby offering a clinically useful tool for risk stratification.

### Statistical analysis

Continuous data were showed as either mean (s.d.) or median (IQR 25–75) and compared using the Student’s t test or Mann-Whitney U test. Categorical data were showed as number and proportion and compared using the χ^2^ test.

The discriminatory predictive performances and clinical practicability of the CART strategy and others commonly used staging systems were calculated using time-dependent receiver operating characteristic (t-ROC) curves and decision curve analyses (DCA), respectively. The prediction consistency of CART model was assessed using calibration plots. To confirm the optimal cut-off value for assessing ER risk, X-tile software was used for clinical decision-making. Based on this, all cases were classified as low-, mediate-, and high-risk subgroups. The RFS and OS curves were assessed via the Kaplan-Meier method and compared using the log-rank test.

All statistical analyses were carried out using SPSS (v25.0). All tests were two-tailed, and statistical significance were considered for *P*-values < 0.05.

## Results

### Patients’ characteristics

A total of 440 patients with HCC and MVI who underwent curative-intent liver resection were included in the study. The cohort comprised 384 men and 56 women. Although liver function was preserved in all patients, 383 (87.0%) were hepatitis B virus (HBV)-positive, 224 (50.9%) exhibited elevated HBV-DNA levels, and 218 (49.5%) had underlying cirrhosis.

Regarding tumor burden, 282 patients (64.1%) had larger tumor sizes and 104 (23.6%) presented with multiple tumors. In terms of pathological features, 58.4% exhibited Edmondson-Steiner (ES) grade III or IV, 51.4% had incomplete tumor capsule formation, 59.1% showed tumor necrosis, 12.7% had satellite nodules, 25.2% demonstrated high p53 mutation rates, and 63.0% exhibited a high Ki-67 proliferation index. The optimal cut-off values for p53 mutation rate and Ki-67 index were determined using X-tile software (**Supplementary Figure 2**).

Baseline clinical staging according to various systems is detailed in **Supplementary Table 1**. Based on MVI classification, 329 patients (74.8%) were categorized into the low-risk group (M1), and 111 (25.2%) into the high-risk group (M2). Significant differences between the two groups were observed in several clinicopathologic variables, including HBV-DNA load, prealbumin (PA), aspartate aminotransferase (AST), tumor size, tumor number, ES grade, presence of satellite nodules, tumor necrosis, and Ki-67 index (**Table 1**). Differences in BCLC and TNM staging distributions were also noted (**Supplementary Table 1**).

**Table 1:** Clinicopathological features of included patients between low and high risk of MVI.

Variables	Total (n = 440)	M1 (n = 329)	M2 (n = 111)	*P* value
Age, years	51 ± 11	51 ± 11	51 ± 11	0.916
Sex				0.774
Male	384 (87.3)	288 (87.5)	96 (86.5)	
Female	56 (12.7)	41 (12.5)	15 (13.5)	
Positive HBsAg	383 (87.0)	286 (86.9)	97 (87.4)	0.901
HBV-DNA, IU/mL				0.021
< 2000	216 (49.1)	172 (52.3)	44 (39.6)	
≥ 2000	224 (50.9)	157 (47.7)	67 (60.4)	
TBil, μmol/L	13.4 (10.1, 17.3)	13.0 (9.9, 17.3)	14.6 (10.7, 17.8)	0.427
PA, mg/L	183.0 (147.0, 223.0)	188.5 (150.0, 230.0)	167.0 (144.0, 201.0)	0.004
ALB, g/L	39.2 (36.6, 42.5)	40.0 (36.6, 43.0)	38.5 (36.6, 40.7)	0.054
ALT, U/L	34.0 (24.0, 47.0)	34.0 (24.0, 46.3)	34.0 (23.5, 50.5)	0.467
AST, U/L	36.0 (29.0, 54.0)	35.0 (29.0, 51.3)	40.0 (30.0, 61.0)	0.011
CR, μmol/L	78.0 (70.0, 87.0)	78.0 (70.0, 87.0)	78.0 (68.0, 88.0)	0.456
PT, s	12.8 (12.1, 13.7)	12.7 (12.1, 13.7)	12.8 (12.0, 13.6)	0.983
Child-Pugh	5 (5, 5)	5 (5, 5)	5 (5, 5)	0.591
MELD	5 (3, 7)	5 (3, 7)	5 (3, 7)	0.716
ALBI	-2.57 (-2.90, -2.36)	-2.64 (-2.93, -2.34)	-2.48 (-2.79, -2.36)	0.058
AFP, ng/mL				0.537
< 400	241 (54.8)	183 (55.6)	58 (52.3)	
≥ 400	199 (45.2)	146 (44.4)	53 (47.7)	
Ascites	41 (9.3)	32 (9.7)	9 (8.1)	0.612
CSPH	31 (7.0)	21 (6.4)	10 (9.0)	0.350
Cirrhosis	218 (49.5)	164 (49.8)	54 (48.6)	0.827
Tumor size, cm				0.043
< 5	158 (35.9)	127 (38.6)	31 (27.9)	
≥ 5	282 (64.1)	202 (61.4)	80 (72.1)	
Tumor number				0.012
Single	336 (76.4)	261 (79.3)	75(67.6)	
Multiple	104 (23.6)	68 (20.7)	36 (32.4)	
ES grade				0.003
I or II	183 (41.6)	150 (45.6)	33 (29.7)	
III or IV	257 (58.4)	179 (54.4)	78 (70.3)	
Tumor capsule				0.663
Complete	214 (48.6)	162 (49.2)	52 (46.8)	
Incomplete	226 (51.4)	167 (50.8)	59 (53.2)	
Satellite nodules	56 (12.7)	30 (9.1)	26 (23.4)	<0.001
Tumor necrosis	260 (59.1)	182 (55.3)	78 (70.3)	0.006
p53 mutation rate				0.077
Low	329 (74.8)	253 (76.9)	76 (68.5)	
High	111 (25.2)	76 (23.1)	35 (31.5)	
Ki67 positive index				0.004
Low	163 (37.0)	133 (40.4)	28 (25.2)	
High	277 (63.0)	196 (59.6)	83 (74.8)	
Positive CK19	101 (23.0)	80 (24.3)	29 (26.1)	0.702
Resection margin, cm				0.922
< 1	366 (83.2)	274 (83.3)	92 (83.9)	
≥ 1	74 (16.8)	55 (16.7)	19 (17.1)	
Operation time, min	190 (150, 225)	190 (155, 226)	190 (150, 223)	0.534
Blood loss, mL				0.618
< 400	321 (73.0)	238 (72.3)	83 (74.8)	
≥ 400	119 (27.0)	91 (27.7)	28 (25.2)	
Blood transfusion	56 (12.7)	40 (12.2)	16 (14.4)	0.537

Data are median (IQR 25–75) unless otherwise indicated.

HCC, hepatocellular carcinoma; HBsAg hepatitis B surface antigen; HBV-DNA hepatitis B virus DNA load; T-Bil, total bilirubin; PA, prealbumin; ALB, albumin; ALT, alanine transaminase; AST, aspartic aminotransferase; CR, creatinine; PT, prothrombin time; MELD, model for end-stage liver disease; ALBI, albumin–bilirubin; AFP, α-fetoprotein; CSPH, clinically significant portal hypertension; ES, Edmondson-Steiner; MVI, microvascular invasion; CK19, cytokeratin 19.

Patients were randomly allocated to the training cohort (n=332) and the validation cohort (n=108) in a 3:1 ratio. No significant differences in baseline characteristics were observed between the two cohorts (**Table 2**; *P* > 0.05 for all variables). Clinical staging distributions for both cohorts are shown in **Supplementary Table 2**.

**Table 2:** Clinicopathological features of the training cohort and validation cohort.

Variables	Training cohort (n=332)	Validation cohort (n=108)	*P* value
Age, years	50 ± 11	53 ± 10	0.132
Sex			0.361
Male	287 (86.4)	97 (89.8)	
Female	45 (13.6)	11 (10.2)	
Positive HBsAg	288 (86.7)	95 (88.0)	0.744
HBV-DNA, IU/mL			0.504
< 2000	166 (50.0)	50 (46.3)	
≥ 2000	166 (50.0)	58 (53.7)	
TBil, μmol/L	13.6 (10.2, 17.7)	12.9 (9.7, 17.3)	0.113
PA, mg/L	182.0 (147.3, 220.8)	183.0 (139.0, 230.3)	0.383
ALB, g/L	39.1 (36.5, 42.6)	39.2 (36.8, 41.8)	0.783
ALT, U/L	33.5 (23.0, 46.0)	35.0 (26.0, 54.0)	0.337
AST, U/L	35.5 (29.0, 50.0)	347.0 (30.0, 61.0)	0.168
CR, μmol/L	78.0 (70.0, 88.0)	78.0 (68.0, 85.0)	0.944
PT, s	12.8 (12.0, 13.7)	12.6 (12.1, 13.8)	0.877
Child-Pugh	5 (5, 5)	5 (5, 6)	0.840
MELD	5 (3, 7)	4 (3, 7)	0.291
ALBI	-2.57 (-2.91, -2.35)	-2.58 (-2.88, -2.42)	0.880
AFP, ng/mL			0.973
< 400	182 (54.8)	59 (54.6)	
≥ 400	150 (45.2)	49 (45.4)	
Ascites	29 (8.7)	12 (11.1)	0.461
CSPH	22 (6.6)	9 (8.3)	0.547
Cirrhosis	163 (49.1)	55 (50.9)	0.741
Tumor size, cm			0.778
< 5	118 (35.5)	40 (37.0)	
≥ 5	214 (64.5)	68 (63.0)	
Tumor number			0.519
Single	256 (77.1)	80 (74.1)	
Multiple	76 (22.9)	28 (25.9)	
ES grade			0.253
I or II	133 (40.1)	50 (46.3)	
III or IV	199 (59.9)	58 (53.7)	
Tumor capsule			0.221
Complete	167 (50.3)	47 (43.5)	
Incomplete	165 (49.7)	61 (56.5)	
MVI grade			0.053
M1	243 (73.2)	89 (82.4)	
M2	89 (26.8)	19 (17.6)	
Satellite nodules	41 (12.3)	15 (13.9)	0.677
Tumor necrosis	192 (57.8)	68 (63.0)	0.346
p53 mutation rate			0.655
Low	250 (75.3)	79 (73.1)	
High	82 (24.7)	29 (26.9)	
Ki67 positive index			0.108
Low	130 (39.2)	33 (30.6)	
High	202 (60.8)	75 (69.4)	
Positive CK19	74 (22.3)	27 (25.0)	0.561
Resection margin, cm			0.084
< 1	282 (84.9)	84 (77.8)	
≥ 1	50 (15.1)	24 (22.2)	
Operation time, min	190 (150, 221)	190 (160, 235)	0.673
Blood loss, mL			0.424
< 400	239 (72.0)	82 (75.9)	
≥ 400	93 (28.0)	26 (24.1)	
Blood transfusion	43 (13.0)	13 (12.0)	0.804

Data are median (IQR 25–75) unless otherwise indicated.

HCC, hepatocellular carcinoma; HBsAg hepatitis B surface antigen; HBV-DNA hepatitis B virus DNA load; T-Bil, total bilirubin; PA, prealbumin; ALB, albumin; ALT, alanine transaminase; AST, aspartic aminotransferase; CR, creatinine; PT, prothrombin time; MELD, model for end-stage liver disease; ALBI, albumin-bilirubin; AFP, α-fetoprotein; CSPH, clinically significant portal hypertension; ES, Edmondson-Steiner; MVI, microvascular invasion; CK19, cytokeratin 19.

### Independent risk factors of ER

Among the entire cohort, 222 patients (50.4%) developed ER: 190 had intrahepatic recurrence, 10 had extrahepatic recurrence, and 22 had concurrent intra- and extrahepatic recurrence. ER occurred in 170 of 332 patients (51.2%) in the training cohort and in 52 of 108 patients (48.1%) in the validation cohort.

In the training cohort, univariate Cox regression identified several factors significantly associated with ER, including HBV-DNA load, alpha-fetoprotein (AFP) level, tumor size, ES grade, satellite nodules, tumor capsule status, MVI classification, p53 mutation rate, Ki-67 index, CK19 expression, and intraoperative blood loss (**Table 3**; all *P* < 0.05).

**Table 3:** Univariable and multivariate Cox-regression analyses of prognostic factors affecting early recurrence in HCC patients with MVI after curative hepatectomy in the training cohort.

Variables	Univariable Cox regression		Multivariable Cox regression	
	HR (95%CI)	*P* value	HR (95%CI)	*P* value
Age, years	0.992 (0.978, 1.006)	0.269		
Male Sex	1.189 (0.746, 1.896)	0.468		
Positive HBsAg	1.626 (0.957, 2.763)	0.073		
HBV-DNA ≥ 2000 IU/mL	1.645 (1.209, 2.239)	0.002	1.444 (1.042, 2.001)	0.027
Child-Pugh	1.083 (0.858, 1.366)	0.503		
MELD	1.009 (0.967, 1.052)	0.684		
ALBI	1.096 (0.835, 1.438)	0.510		
AFP ≥ 400 ng/mL	1.390 (1.029, 1.878)	0.032	1.018 (0.731, 1.418)	0.916
Ascites	0.902 (0.522, 1.559)	0.711		
CSPH	1.353 (0.796, 2.298)	0.264		
Cirrhosis	0.992 (0.734, 1.340)	0.957		
Tumor size ≥ 5 cm	1.735 (1.239, 2.430)	0.001	1.568 (1.094, 2.247)	0.014
Multiple tumor number	1.089 (0.759, 1.562)	0.644		
ES (grade III or IV)	1.841 (1.332, 2.546)	<0.001	1.440 (1.024, 2.026)	0.036
Tumor capsule (incomplete)	1.553 (1.147, 2.102)	0.004	1.533 (1.109, 2.119)	0.010
MVI grade (M2)	1.898 (1.386, 2.599)	<0.001	1.420 (1.017, 1.984)	0.040
Satellite nodules	1.988 (1.352, 2.922)	<0.001	1.617 (1.071, 2.439)	0.022
Tumor necrosis	1.330 (0.976, 1.812)	0.071		
High p53 mutation rate	1.546 (1.109, 2.155)	0.010	1.045 (0.712, 1.533)	0.822
High Ki67 positive index	1.778 (1.284, 2.461)	0.001	1.422 (1.008, 2.006)	0.045
Positive CK19	1.774 (1.275, 2.470)	0.001	1.423 (1.010, 2.011)	0.047
Resection margin ≥ 1 cm	1.228 (0.802, 1.879)	0.345		
Operation time, min	1.001 (0.998, 1.003)	0.525		
Blood loss ≥ 400 mL	1.407 (1.023, 1.935)	0.036	1.168 (0.822, 1.658)	0.386
Blood transfusion	1.380 (0.889, 2.144)	0.151		

HCC, hepatocellular carcinoma; HBsAg hepatitis B surface antigen; HBV-DNA hepatitis B virus DNA load; MELD, model for end-stage liver disease; ALBI, albumin-bilirubin; AFP, α-fetoprotein; CSPH, clinically significant portal hypertension; ES, Edmondson-Steiner; MVI, microvascular invasion; CK19, cytokeratin 19.

Multivariate Cox regression analysis further identified HBV-DNA load, tumor size, ES grade, incomplete tumor capsule, MVI classification, presence of satellite nodules, Ki-67 index, and positive CK19 expression as independent predictors of ER (**Table 3**; *P* < 0.05 for all). The RFS curves stratified by these risk factors are presented in **Supplementary Figure 3**.

### Construction and validation of the CART Model

**Figure 1:**
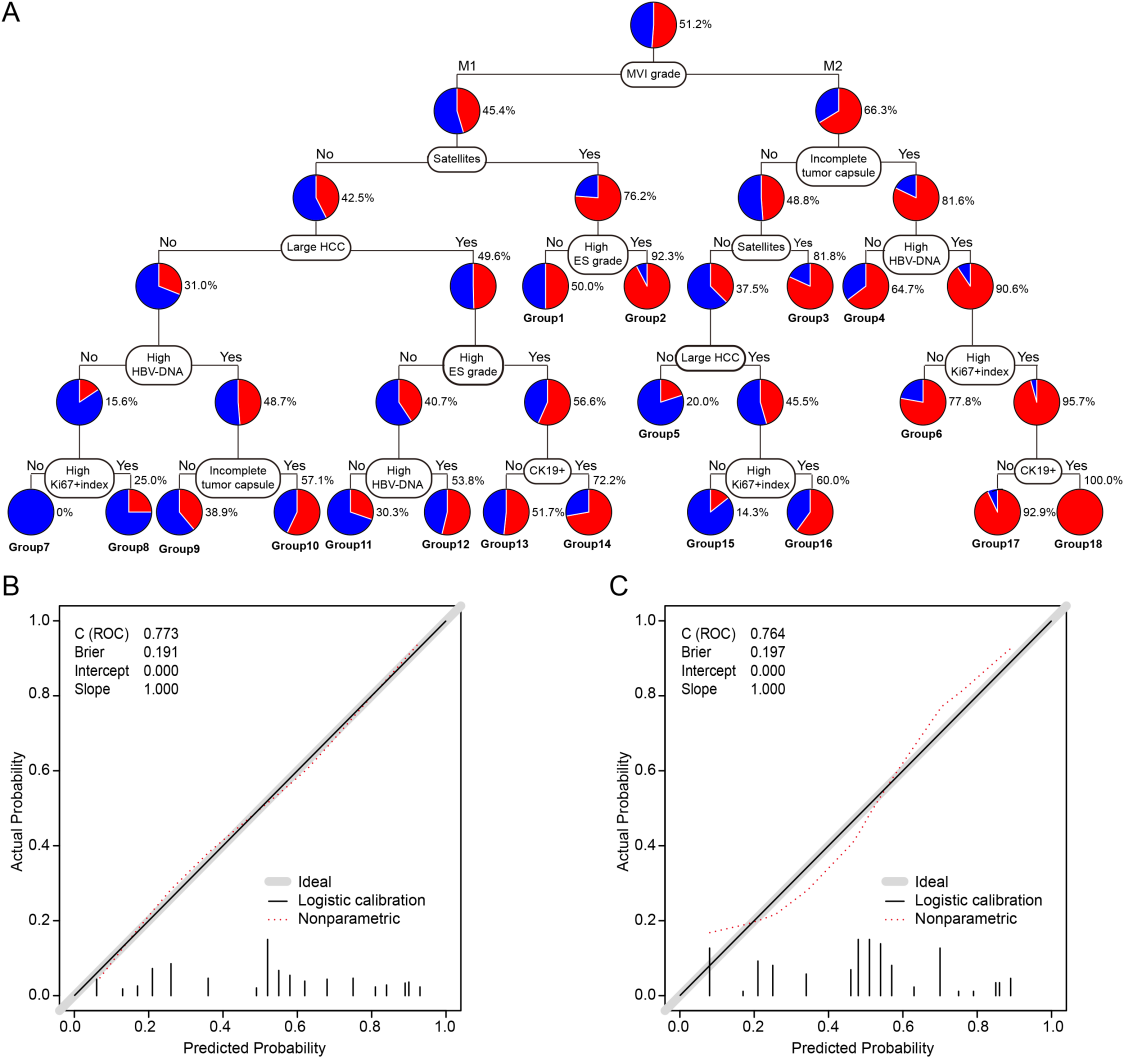
Construction and calibration of the CART model for predicting early recurrence in HCC patients with MVI. **(A)** CART model for ER prediction after curative hepatectomy. Terminal patient groups were numbered from 1 to 18. **(B)** Calibration plot for the training cohort. **(C)** Calibration plot for the validation cohort. CART, classification and regression tree; ER, early recurrence; HCC, hepatocellular carcinoma; MVI, microvascular invasion; HBV-DNA, hepatitis B virus DNA load; Ki-67, proliferation marker protein Ki-67; CK19, cytokeratin 19.

The eight independent prognostic factors identified for ER were incorporated into a CART model. The resulting decision tree is illustrated in **Figure 1A**. MVI classification served as the first node, followed by tumor size, Ki-67 index, and other variables, allowing stratification of patients into distinct ER risk subgroups.

The C-statistic for the CART model in predicting ER was 0.773 (95% confidence interval [CI]: 0.724–0.822) in the training cohort, with calibration plots demonstrating good agreement between predicted and observed outcomes (**Figure 1B**). In the validation cohort, the C-statistic was 0.764 (95% CI: 0.674–0.854), and calibration analysis similarly indicated good model fit (**Figure 1C**).

### Comparison of CART and conventional staging systems in predicting ER

**Figure 2:**
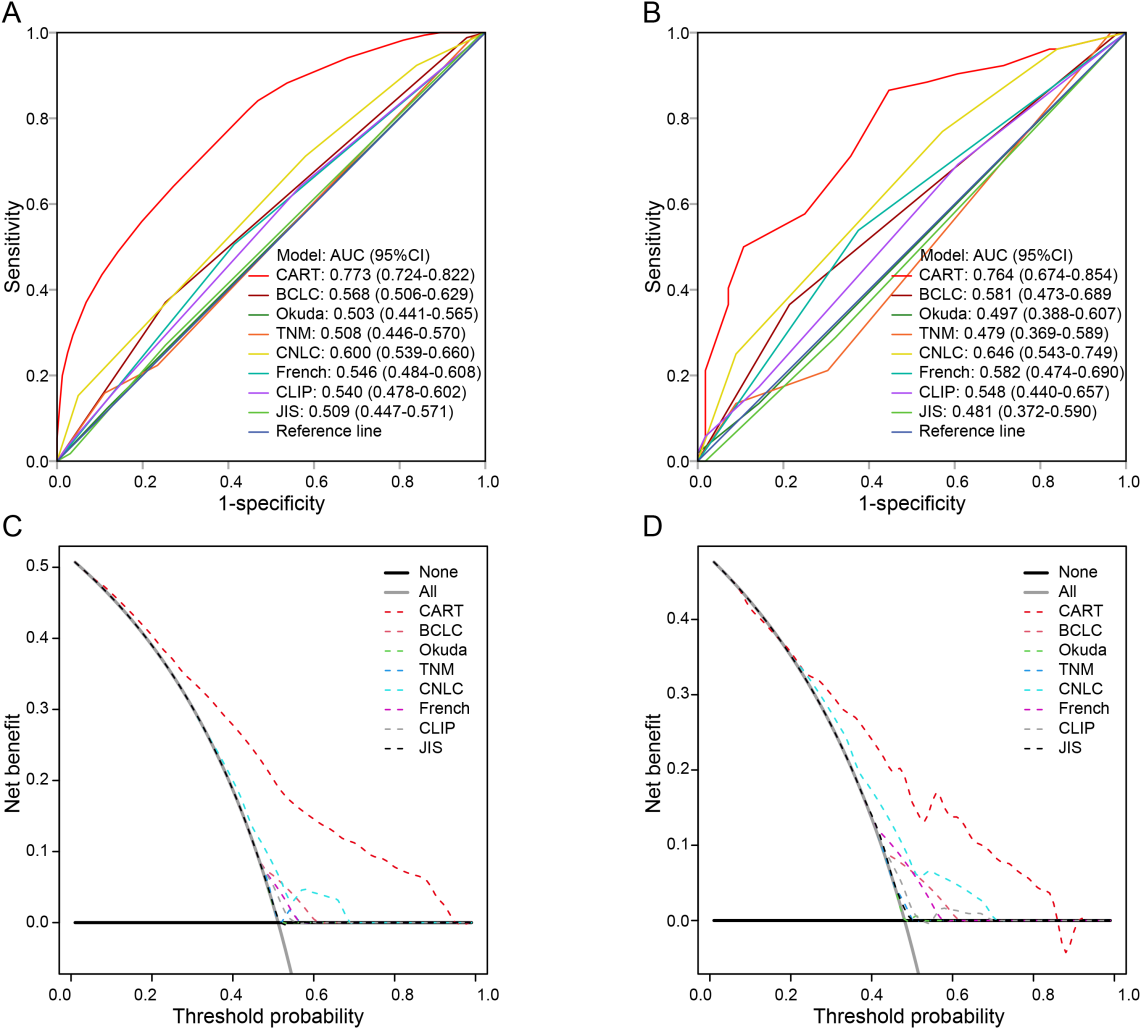
Comparison of the CART model and conventional staging systems for predicting early recurrence. **(A, B)** Time-dependent ROC curves in the training and validation cohorts, respectively. **(C, D)** DCA for the CART model and traditional staging systems in the training and validation cohorts. ER, early recurrence; HCC, hepatocellular carcinoma; MVI, microvascular invasion; CART, classification and regression tree; BCLC, Barcelona Clinic Liver Cancer; TNM, tumor-node-metastasis; CNLC, China Liver Cancer staging system; CLIP, Cancer of the Liver Italian Program; JIS, Japan Integrated Staging; ROC, receiver operating characteristic; DCA, decision curve analysis.

The discriminatory ability and clinical utility of the CART model were compared with traditional staging systems. As shown in **Figure 2A**, in the training cohort, the area under the curve (AUC) of the CART model (0.773) was significantly higher than those of the BCLC, TNM, CNLC, French, Okuda, CLIP, and JIS staging systems (AUC range: 0.503–0.600; all *P* < 0.05). Similar findings were observed in the validation cohort (**Figure 2B**).

Decision curve analysis (DCA) further demonstrated that the CART model provided superior net benefit across a wide range of threshold probabilities compared with conventional staging systems (**Figures 2C, D**). Detailed AUC values are provided in **Supplementary Table 3**.

### Performance of CART in predicting RFS and OS

In the training and validation cohorts, the median follow-up duration for RFS was 10.0 (interquartile range [IQR]: 3–24) and 11.5 (IQR: 3–30) months, respectively. Recurrence occurred in 195 (58.7%) and 55 (50.9%) patients, respectively. The 1-, 2-, and 3-year RFS rates were 49.9%, 34.6%, and 27.2% in the training cohort, and 48.7%, 36.9%, and 33.4% in the validation cohort (P > 0.05 for all comparisons).

**Figure 3:**
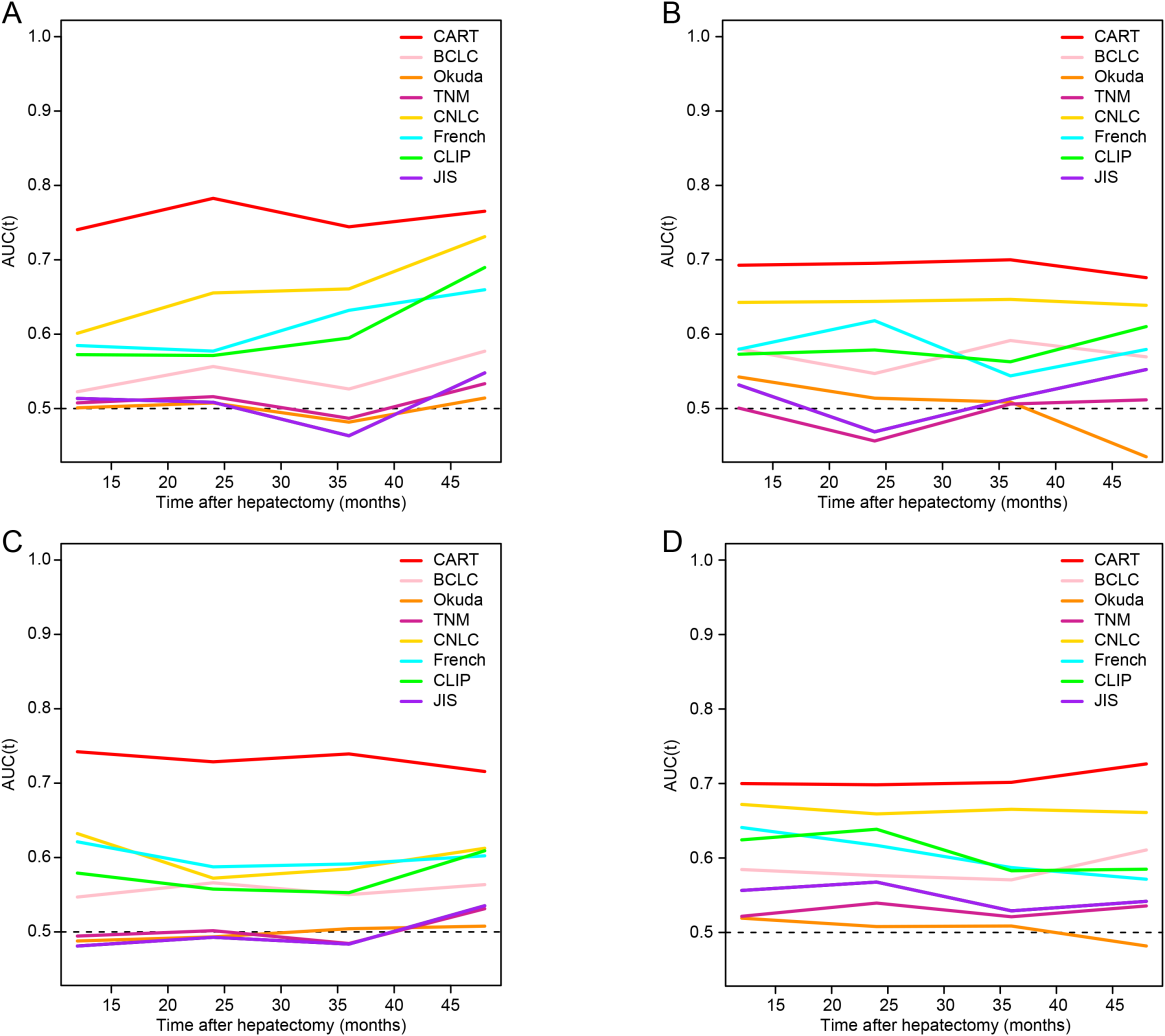
Time-dependent ROC analysis for recurrence-free survival and overall survival. **(A, B)** Time-dependent ROC curves for RFS in the training and validation cohorts, respectively. **(C, D)** Time-dependent ROC curves for OS in the training and validation cohorts. RFS, recurrence-free survival; OS, overall survival; HCC, hepatocellular carcinoma; MVI, microvascular invasion; CART, classification and regression tree; BCLC, Barcelona Clinic Liver Cancer; TNM, tumor-node-metastasis; CNLC, China Liver Cancer staging system; CLIP, Cancer of the Liver Italian Program; JIS, Japan Integrated Staging; ROC, receiver operating characteristic.

The CART model achieved consistently higher AUC values at 1-, 2-, and 3-year time points compared to other staging systems in both cohorts (**Figures 3A, B**). Corresponding AUC values are detailed in **Supplementary Table 4**.

The median follow-up for OS was 40 (IQR: 12–60) months in the training cohort and 37 (IQR: 12–59) months in the validation cohort. Mortality was observed in 117 (35.2%) and 44 (40.7%) patients, respectively. The 1-, 2-, and 3-year OS rates were 80.0%, 69.1%, and 60.6% in the training cohort, and 71.0%, 62.0%, and 50.9% in the validation cohort (*P* > 0.05 for all comparisons).

The CART model consistently outperformed conventional staging systems in OS prediction at all time points (**Figures 3C, D**). AUC results for OS prediction are provided in **Supplementary Table 5**.

### Performance of CART strategy in stratifying Risk

Patients were stratified into three risk groups based on ER probabilities derived from the CART model: low risk (<0.50), intermediate risk (0.50–0.82), and high risk (≥0.82), as determined by X-tile analysis (**Supplementary Figure 4**).

**Figure 4:**
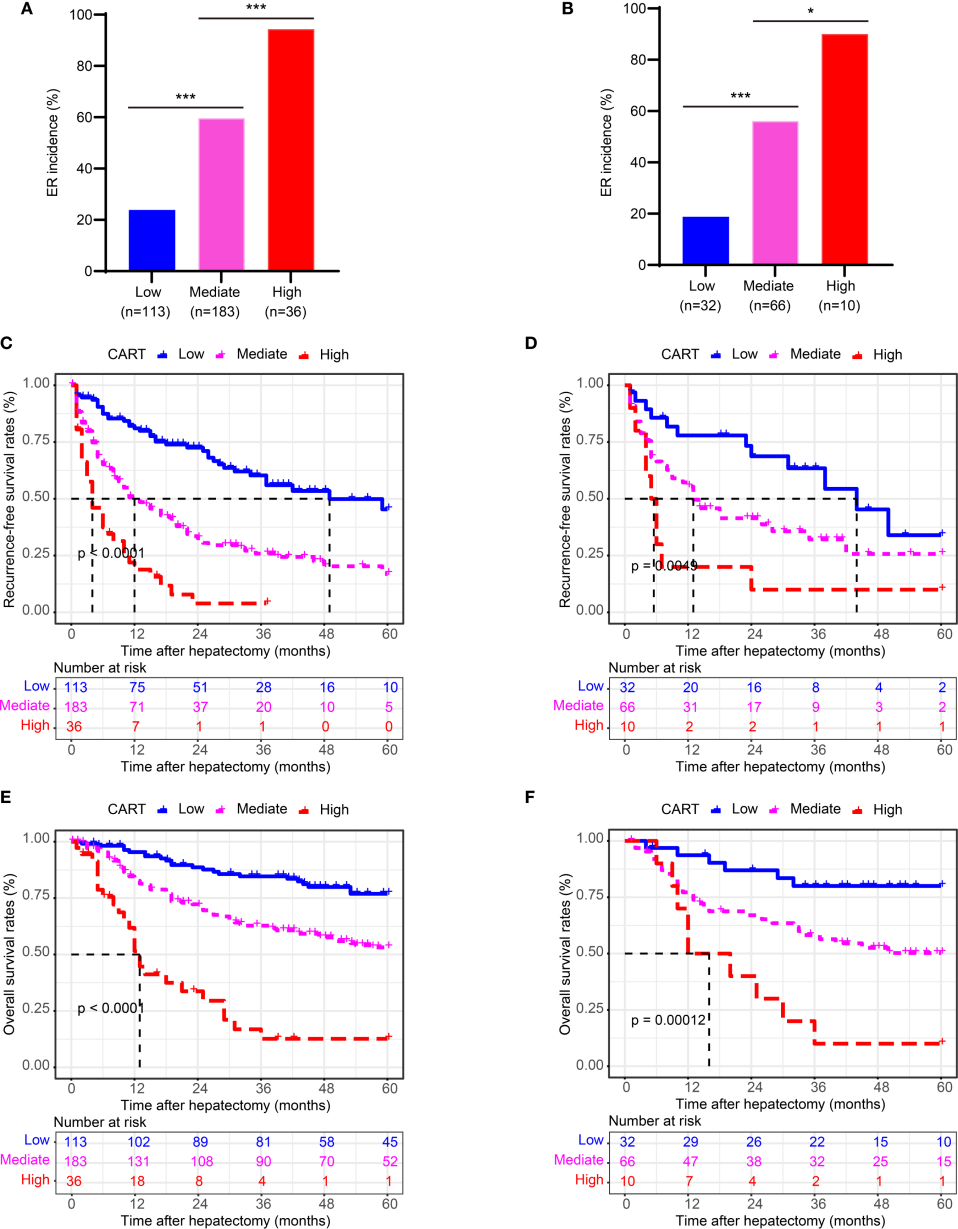
Risk stratification by the CART model for ER, RFS, and OS. **(A, B)** Kaplan-Meier curves of ER stratified by CART-based risk groups in the training and validation cohorts. **(C, D)** Kaplan-Meier curves of RFS in the training and validation cohorts. **(E, F)** Kaplan-Meier curves of OS in the training and validation cohorts. CART, classification and regression tree; ER, early recurrence; RFS, recurrence-free survival; OS, overall survival.

Significant differences in ER incidence were observed among the three risk groups in both the training and validation cohorts (**Figures 4A, B**; *P* < 0.001). Furthermore, stratified analysis revealed significant differences in both RFS and OS among the three groups (**Figures 4C-F**; *P* < 0.001 for all comparisons).

These findings confirm that the CART-based risk stratification effectively discriminates patient outcomes following curative hepatectomy for HCC with MVI.

## Discussion

This study developed and validated a CART model for predicting ER in patients with HCC and MVI who underwent curative resection. Based on clinicopathological and molecular data from 440 patients, the model integrated eight independent predictors—including HBV-DNA load, tumor size, ES grade, tumor capsule integrity, MVI classification, presence of satellite nodules, Ki-67 index, and CK19 expression—and demonstrated excellent discriminative ability (C-index = 0.773 in the training cohort and 0.764 in the validation cohort) and calibration performance. Compared with conventional staging systems such as BCLC, TNM, and CNLC, the CART model achieved higher AUC values and greater clinical net benefit in predicting ER, RFS, and OS, highlighting its utility for risk stratification in postoperative HCC management.

From a mechanistic perspective, our findings further emphasize the central role of MVI in HCC recurrence. MVI is not only a morphological manifestation of tumor cell invasion into vascular structures but also reflects underlying molecular alterations and reprogramming of the tumor microenvironment (TME) (26). As the primary splitting node in the model, MVI classification (M1 vs. M2) directly illustrates the biological continuum between the extent of invasion and early recurrence risk. High-risk MVI (M2) is often associated with activation of epithelial-mesenchymal transition (EMT), involving downregulation of E-cadherin and upregulation of N-cadherin and vimentin, which enhances tumor cell migration and invasiveness (27, 28). Furthermore, MVI-positive tumors frequently exhibit vasculogenic mimicry and lymphatic infiltration tendencies, providing a structural basis for the survival and regeneration of minimal residual disease after surgery (29, 30). Notably, patients with M2-type MVI often presented with larger tumor burden, higher Ki-67 proliferation index, and poorer histological differentiation, suggesting a more aggressive biological phenotype. These results are consistent with the clinical utility of the MVI grading system proposed by Cong et al. (6) and underscore the importance of standardizing MVI classification in pathological reporting.

The liver maintains a uniquely tolerant immune environment, constantly bathed in diverse foreign antigens due to its role as a tolerogenic organ. However, under specific pathological conditions, this state of tolerance can be broken, leading to pathological inflammation and dysregulated immune responses (31, 32). This dual immune role is closely associated with the development of HCC, MVI, and ER. Chronic HBV infection remains a dominant etiological factor for HCC, particularly in Asia (33). In our cohort, 87.0% of patients were HBV-positive. Elevated HBV-DNA load was independently associated with increased ER risk, consistent with prior studies (11, 34). Persistent viral replication may contribute to microenvironmental changes favoring tumor recurrence. These findings underscore the importance of stringent viral suppression to reduce recurrence and improve survival in HBV-related HCC patients. Tumor size is another critical determinant of prognosis (5, 35). Larger tumors often exhibit features such as microvascular infiltration, an incomplete capsule, and satellite nodules, all of which are associated with early dissemination. Our data confirm that larger tumor is significantly associated with a higher risk of ER, supporting the need for more aggressive postoperative surveillance and consideration of adjuvant therapies in this subgroup. Other pathological factors, including a higher ES grade, incomplete tumor capsule, presence of satellite nodules, and MVI subclassification, further stratify recurrence risk (6, 11, 36). We adopted the MVI grading system proposed by Cong et al. (6) due to its simplicity and clinical applicability. High-risk MVI was associated with more aggressive biological features and poorer outcomes, reinforcing its prognostic relevance.

Regarding molecular markers, both Ki-67 and CK19 also emerged as significant prognosticators. Ki-67, a nuclear protein involved in cell proliferation (37), has been consistently associated with poor outcomes in HCC (38, 39). Using X-tile analysis, we identified an optimal threshold for Ki-67 positivity, and higher expression correlated with unfavorable clinicopathological features and worse survival. CK19, a marker of hepatic progenitor cells (40, 41), was similarly linked to aggressive tumor biology and adverse prognosis, particularly in HBV-associated HCC (42). These results suggest that incorporating molecular biomarkers into predictive models can significantly improve the accuracy of risk stratification and reflect deeper immunobiological mechanisms underlying tumor recurrence, including abnormal immune responses caused by dysregulated antigen presentation mechanisms—observed not only in neoplastic diseases but also in non-neoplastic liver conditions such as autoimmune hepatitis (43, 44). Therefore, the predictive model constructed in this study integrates not only clinical and pathological features but also key immunological dimensions of recurrence biology, providing a more comprehensive basis for individualized prognostic assessment and treatment decision-making.

From a methodological standpoint, the advantage of the CART model lies in its ability to capture complex interactions and nonlinear relationships among predictive variables, making it more aligned with real-world clinical decision-making scenarios. For example, among patients with M1-type MVI, the model further stratifies risk based on tumor size and Ki-67 index, identifying subgroups still at high risk of recurrence—suggesting that even with less severe MVI, combined high proliferative activity and large tumor burden should warrant vigilance for early recurrence. For M2-type MVI patients, CK19 expression and satellite nodule status further refine risk classification, demonstrating the importance of multi-parameter integration in prognostic assessment.

This study develops a predictive model for ER following curative HCC resection, addressing a distinct clinical challenge that differs from initial diagnosis or general surveillance paradigms. While established serum biomarkers (AFP and DCP) remain indispensable for HCC surveillance and diagnosis per international guidelines (2, 3, 45), their post-operative predictive utility is constrained by variable sensitivity/specificity, particularly when values normalize post-resection (46, 47). Our perioperative CART model addresses this gap by complementing—rather than replacing—existing biomarker monitoring and imaging protocols. Designed for immediate surgical or post-operative risk stratification, the algorithm identifies high-risk patients before conventional surveillance detects recurrence, enabling tailored follow-up intensity. High-risk patients could receive intensified monitoring despite unremarkable initial AFP/DCP levels, while low-risk cohorts might adopt reduced surveillance frequency, optimizing resource allocation and minimizing patient distress (48). By augmenting current strategies with this prognostication layer, the model aims to expedite recurrence detection and therapeutic intervention, potentially improving long-term outcomes through personalized surveillance adaptation.

Despite the encouraging findings, this study also has several limitations. First, the retrospective single-center design may introduce selection bias. Although internal validation was conducted, external validation across multi-center cohorts involving diverse etiologies, such as non-HBV-related HCC, is needed to verify the model’s generalizability. Second, while the CART model incorporated several key biological predictors, it did not include emerging dynamic biomarkers like ctDNA or quantitative radiomic features, which may further enhance the accuracy of risk stratification. Subsequent studies could integrate multi-omics data to refine the model and strengthen its predictive capability. Moreover, the study only enrolled patients who underwent curative resection, which may introduce selection bias and restrict the applicability of the conclusions to surgical candidates with early-stage disease. Therefore, the results should not be generalized to patients with advanced HCC or those ineligible for surgical intervention. In addition, due to the retrospective nature of the study, post-recurrence treatment strategies were highly individualized and inconsistently documented, hindering systematic collection and analysis of postoperative management data. As a result, treatment-related variables were excluded from covariate adjustment and subgroup analyses. Future studies should account for heterogeneity in adjuvant therapies as a crucial factor to allow a more comprehensive evaluation of recurrence risk. Finally, although CART offers clinical interpretability, future work may explore hybrid models combining interpretable tree-based frameworks with more complex machine learning approaches to optimize both transparency and predictive accuracy.

## Conclusion

The development and validation of an MVI-based CART strategy has provided a valuable tool for predicting ER risk, RFS, and OS in patients with HCC. This strategy may assist clinicians in tailoring postoperative surveillance programs and therapeutic interventions for HCC patients with MVI following curative hepatectomy.

## Data Availability

The raw data supporting the conclusions of this article will be made available by the authors, without undue reservation.
