# Supplementary material for: A CART-based prognostic model for risk stratification of postoperative early recurrence in hepatocellular carcinoma with microvascular invasion
Source: Front Oncol. 2025 Oct 24;15:1655739. doi: 10.3389/fonc.2025.1655739 (PMC12591875; doi:10.3389/fonc.2025.1655739)
Supplement: Supplementary file 1 [file DataSheet1.docx]

**Supplementary Materials for:**

**Risk Stratification for Postoperative Early Recurrence in Hepatocellular Carcinoma with Microvascular Invasion Using a CART-Based Prognostic Model**

**Authors:**

Jie Zeng, M.D.¹²*; Ri-Jin Lu, M.M.¹³*; Zheng Tao, M.M.¹³; Can Zeng, M.M.¹³; Kai-Xiang Mo, M.M.¹³; Wei-Jie Cen, M.M.¹³; Yan Lin, M.D.⁵³; Rong Liang, M.D.⁵³; Le-Qun Li, M.D.¹³; Guo-Bin Wu, M.D.¹³^#^; Jia-Zhou Ye, M.D.¹³^#^; Rong-Yun Mai, M.D.¹³^#^

**Contents:**

[Supplementary Table 1. Comparison of Patient Distributions Across Staging Systems Between Low- and High-Risk MVI Groups. 1](#_Toc2029)

[Supplementary Table 2. Distribution of Patients by Staging Systems in Training and Validation Cohorts. 2](#_Toc16503)

[Supplementary Table 3. Discriminatory performance of the CART model and other staging systems in predicting ER in HCC patients with MVI after curative hepatectomy in training and validation cohort. 3](#_Toc27624)

[Supplementary Table 4. Comparison of different Staging Systems to predict postoperative 1-year, 2-year and 3-year RFS in HCC patients with MVI in both training and validation cohorts. 4](#_Toc30874)

[Supplementary Table 5. Comparison of different Staging Systems to predict postoperative 1-year, 2-year, and 3-year OS in HCC patients with MVI in both training and validation cohorts. 5](#_Toc15072)

[Supplementary Figure 1. Flowchart illustrating patient selection, exclusion criteria, and cohort allocation used for CART model development and validation. 7](#_Toc15019)

[Supplementary Figure 2. Optimal cut-off values were defined using X-tile software. 8](#_Toc10344)

[Supplementary Figure 3. Recurrence-risk stratification for eight prognostic variables in the training cohort.. 9](#_Toc5383)

[Supplementary Figure 4. Risk group classification using the CART model based on cut-off values determined by X-tile software. 10](#_Toc30228)

**Supplementary Table 1.** Comparison of Patient Distributions Across Staging Systems Between Low- and High-Risk MVI Groups.

| Staging systems | Total (n=440) | Low-Risk (n=329) | High-Risk (n=111) | *P* value |
| --- | --- | --- | --- | --- |
| BCLC stage |  |  |  | **0.043** |
| 0 | 10 (2.3) | 7 (2.1) | 3 (2.7) |  |
| A | 295 (67.0) | 231 (70.2) | 64 (57.7) |  |
| B | 135 (30.7) | 91 (27.7) | 44 (39.6) |  |
| TNM stage |  |  |  | **0.024** |
| I | 336 (76.4) | 261 (79.3) | 75 (67.6) |  |
| II | 47 (10.7) | 33 (10.0) | 14 (12.6) |  |
| III | 57 (13.0) | 35 (10.6) | 22 (19.8) |  |
| Okuda stage |  |  |  | 0.256 |
| I | 385 (87.5) | 287 (87.2) | 98 (88.3) |  |
| II | 54 (12.3) | 42 (12.8) | 12 (10.8) |  |
| III | 1 (0.2) | 0 (0) | 1 (0.9) |  |
| CNLC stage |  |  |  | **0.022** |
| Ia | 50 (11.4) | 38 (11.6) | 12 (10.8) |  |
| Ib | 103 (23.4) | 85 (25.8) | 18 (16.2) |  |
| IIa | 235 (53.4) | 175 (53.2) | 60 (54.1) |  |
| IIb | 52 (11.8) | 31 (9.4) | 21 (18.9) |  |
| French stage |  |  |  | 0.503 |
| A | 238 (54.1) | 181 (55.0) | 57 (51.4) |  |
| B | 202 (45.9) | 148 (45.0) | 54 (48.6) |  |
| CLIP score |  |  |  | 0.200 |
| 0 | 169 (38.4) | 133 (40.4) | 36 (32.4) |  |
| 1 | 204 (46.4) | 151 (45.9) | 53 (47.7) |  |
| 2 | 53 (12.0) | 36 (10.9) | 17 (15.3) |  |
| 3 | 13 (3.0) | 9 (2.7) | 4 (3.6) |  |
| 4 | 1 (0.2) | 0 (0) | 1 (0.9) |  |
| JS score |  |  |  | 0.065 |
| 0 | 3 (0.7) | 2 (0.6) | 1 (0.9) |  |
| 1 | 317 (72.0) | 247 (75.1) | 70 (63.1) |  |
| 2 | 111 (25.2) | 73 (22.2) | 38 (34.2) |  |
| 3 | 9 (2.0) | 7 (2.1) | 2 (1.8) |  |

*Abbreviations: BCLC, Barcelona Clinic Liver Cancer; TNM, tumor-node-metastasis; CNLC, China Liver Cancer staging system; CLIP, Cancer of the Liver Italian Program; JIS, Japan Integrated Staging; MVI, microvascular invasion.*

**Supplementary Table 2.** Distribution of Patients by Staging Systems in Training and Validation Cohorts.

| Staging systems | Training cohort (n = 332) | Validation cohort (n = 108) | *P* value |
| --- | --- | --- | --- |
| BCLC stage |  |  | 0.458 |
| 0 | 9 (2.7) | 1 (0.9) |  |
| A | 219 (66.0) | 76 (70.4) |  |
| B | 104 (31.3) | 31 (28.7) |  |
| TNM stage |  |  | 0.251 |
| I | 256 (77.1) | 80 (74.1) |  |
| II | 31 (9.3) | 16 (14.8) |  |
| III | 45 (13.6) | 12 (11.1) |  |
| Okuda stage |  |  | 0.206 |
| I | 292 (88.0) | 93 (86.1) |  |
| II | 40 (12.0) | 14 (13.0) |  |
| III | 0 (0) | 1 (0.9) |  |
| CNLC stage |  |  | 0.342 |
| Ia | 39 (11.7) | 11 (10.2) |  |
| Ib | 78 (23.5) | 25 (23.1) |  |
| IIa | 181 (54.5) | 54 (50.0) |  |
| IIb | 34 (10.2) | 18 (16.7) |  |
| French stage |  |  | 0.897 |
| A | 179 (53.9) | 59 (54.6) |  |
| B | 153 (46.1) | 49 (45.4) |  |
| CLIP score |  |  | 0.448 |
| 0 | 131 (39.5) | 38 (35.2) |  |
| 1 | 151 (45.5) | 53 (49.1) |  |
| 2 | 40 (12.0) | 13 (12.0) |  |
| 3 | 10 (3.0) | 3 (2.8) |  |
| 4 | 0 (0) | 1 (0.9) |  |
| JS score |  |  | 0.378 |
| 0 | 3 (0.9) | 0 (0) |  |
| 1 | 242 (72.9) | 75 (69.4) |  |
| 2 | 79 (23.8) | 32 (29.6) |  |
| 3 | 8 (2.4) | 1 (0.9) |  |

*Abbreviations: CART, classification and regression tree; BCLC, Barcelona Clinic Liver Cancer; TNM, tumor-node-metastasis, AJCC 8th Chinese stage (2017 edition); CNLC, China Liver Cancer stage system; CLIP, Cancer of the Liver Italian Program; JIS, Japan Integrated staging.*

**Supplementary Table 3.** Discriminatory performance of the CART model and other staging systems in predicting ER in HCC patients with MVI after curative hepatectomy in training and validation cohort.

| Staging System | Training cohort | | | Validation cohort | | |
| --- | --- | --- | --- | --- | --- | --- |
|  | AUC | 95 % CI | *P* value | AUC | 95 % CI | *P* value |
| **CART model** | **0.773** | **0.724 - 0.822** | **< 0.001** | **0.764** | **0.674 - 0.854** | **< 0.001** |
| BCLC stage | 0.568 | 0.506 - 0.629 | **0.032** | 0.581 | 0.473 - 0.689 | 0.146 |
| Okuda grade | 0.503 | 0.441 - 0.565 | 0.922 | 0.497 | 0.388 - 0.607 | 0.961 |
| AJCC stage | 0.508 | 0.446 - 0.570 | 0.797 | 0.479 | 0.369 - 0.589 | 0.708 |
| CNLC stage | 0.600 | 0.539 - 0.660 | **0.002** | 0.646 | 0.543 - 0.749 | **0.009** |
| French grade | 0.546 | 0.484 - 0.608 | 0.146 | 0.582 | 0.474 - 0.690 | 0.143 |
| CLIP score | 0.540 | 0.478 - 0.602 | 0.205 | 0.548 | 0.440 - 0.657 | 0.386 |
| JIS score | 0.509 | 0.447 - 0.571 | 0.774 | 0.481 | 0.372 - 0.590 | 0.733 |

*Abbreviations: ER, early recurrence; CART, classification and regression tree; BCLC, Barcelona Clinic Liver Cancer; TNM, tumor-node-metastasis, AJCC 8th Chinese stage (2017 edition); CNLC, China Liver Cancer stage system; CLIP, Cancer of the Liver Italian Program; JIS, Japan Integrated staging.*

**Supplementary Table 4.** Comparison of different Staging Systems to predict postoperative 1-year, 2-year and 3-year RFS in HCC patients with MVI in both training and validation cohorts.

| Staging System | Training cohort | | | Validation cohort | | |
| --- | --- | --- | --- | --- | --- | --- |
|  | 1-year RFS | 2-year RFS | 3-year RFS | 1-year RFS | 2-year RFS | 3-year RFS |
| **CART model** | **0.75 (0.69, 0.80)** | **0.80 (0.74, 0.86)** | **0.77 (0.69, 0.85)** | **0.69 (0.58, 0.80)** | **0.70 (0.58, 0.81**) | **0.70 (0.56, 0.84)** |
| BCLC stage | 0.53 (0.47, 0.58) | 0.56 (0.50, 0.62) | 0.52 (0.45, 0.60) | 0.58 (0.49, 0.67) | 0.55 (0.44, 0.65) | 0.59 (0.49, 0.69) |
| Okuda grade | 0.50 (0.46, 0.54) | 0.51 (0.47, 0.55) | 0.48 (0.43, 0.54) | 0.54 (0.47, 0.62) | 0.51 (0.43, 0.59) | 0.51 (0.42, 0.60) |
| AJCC stage | 0.51 (0.46, 0.55) | 0.52 (0.47, 0.57) | 0.50 (0.44, 0.57) | 0.49 (0.40, 0.58) | 0.44 (0.34, 0.55) | 0.48 (0.37, 0.60) |
| CNLC stage | 0.60 (0.54, 0.66) | 0.66 (0.59, 0.72) | 0.66 (0.58, 0.74) | 0.64 (0.54, 0.75) | 0.64 (0.53, 0.76) | 0.65 (0.48, 0.81) |
| French grade | 0.59 (0.53, 0.64) | 0.58 (0.51, 0.64) | 0.63 (0.55, 0.70) | 0.58 (0.48, 0.68) | 0.62 (0.51, 0.73) | 0.54 (0.41, 0.68) |
| CLIP score | 0.58 (0.51, 0.64) | 0.58 (0.51, 0.65) | 0.60 (0.51, 0.69) | 0.57 (0.46, 0.68) | 0.58 (0.46, 0.70) | 0.56 (0.43, 0.70) |
| JIS score | 0.52 (0.47, 0.57) | 0.53 (0.46, 0.58) | 0.48 (0.41, 0.55) | 0.53 (0.44, 0.63) | 0.47 (0.36, 0.58) | 0.51 (0.40, 0.63) |

*Abbreviations: RFS, recurrence-free survival; CART, classification and regression tree; BCLC, Barcelona Clinic Liver Cancer; TNM, tumor-node-metastasis, AJCC 8th Chinese stage (2017 edition); CNLC, China Liver Cancer stage system; CLIP, Cancer of the Liver Italian Program; JIS, Japan Integrated staging. CART score refers to the predicted ER probability derived from the CART model’s terminal nodes.*

**Supplementary Table 5.** Comparison of different Staging Systems to predict postoperative 1-year, 2-year, and 3-year OS in HCC patients with MVI in both training and validation cohorts.

| Staging System | Training cohort | | | Validation cohort | | |
| --- | --- | --- | --- | --- | --- | --- |
|  | 1-year OS | 2-year OS | 3-year OS | 1-year OS | 2-year OS | 3-year OS |
| **CART model** | **0.74 (0.66, 0.82)** | **0.73 (0.66, 0.79)** | **0.74 (0.68, 0.80)** | **0.70 (0.56, 0.83)** | **0.69 (0.47, 0.81)** | **0.69 (0.58, 0.80)** |
| BCLC stage | 0.55 (0.47, 0.63) | 0.57 (0.50, 0.63) | 0.55 (0.49, 0.61) | 0.58 (0.47, 0.70) | 0.58 (0.48, 0.68) | 0.57 (0.48, 0.66) |
| Okuda grade | 0.49 (0.44, 0.54) | 0.49 (0.45, 0.53) | 0.50 (0.46, 0.54) | 0.52 (0.43, 0.61) | 0.51 (0.44, 0.58) | 0.51 (0.44, 0.58) |
| AJCC stage | 0.48 (0.42, 0.55) | 0.49 (0.44, 0.55) | 0.47 (0.43, 0.53) | 0.54 (0.43, 0.65) | 0.55 (0.45, 0.65) | 0.53 (0.44, 0.62) |
| CNLC stage | 0.63 (0.55, 0.72) | 0.57 (0.50, 0.64) | 0.58 (0.52, 0.65) | 0.67 (0.54, 0.81) | 0.66 (0.55, 0.77) | 0.67 (0.57, 0.77) |
| French grade | 0.62 (0.54, 0.70) | 0.59 (0.52, 0.65) | 0.59 (0.53, 0.65) | 0.64 (0.52, 0.76) | 0.61 (0.51, 0.72) | 0.58 (0.48, 0.68) |
| CLIP score | 0.58 (0.50, 0.66) | 0.56 (0.49, 0.63) | 0.55 (0.49, 0.62) | 0.62 (0.48, 0.76) | 0.64 (0.53, 0.75) | 0.58 (0.47, 0.69) |
| JIS score | 0.48 (0.41, 0.55) | 0.49 (0.44, 0.55) | 0.48 (0.43, 0.54) | 0.56 (0.44, 0.67) | 0.57 (0.47, 0.67) | 0.53 (0.44, 0.63) |

*Abbreviations: OS, overall survival; CART, classification and regression tree; BCLC, Barcelona Clinic Liver Cancer; TNM, tumor-node-metastasis, AJCC 8th Chinese stage (2017 edition); CNLC, China Liver Cancer stage system; CLIP, Cancer of the Liver Italian Program; JIS, Japan Integrated staging.*

**
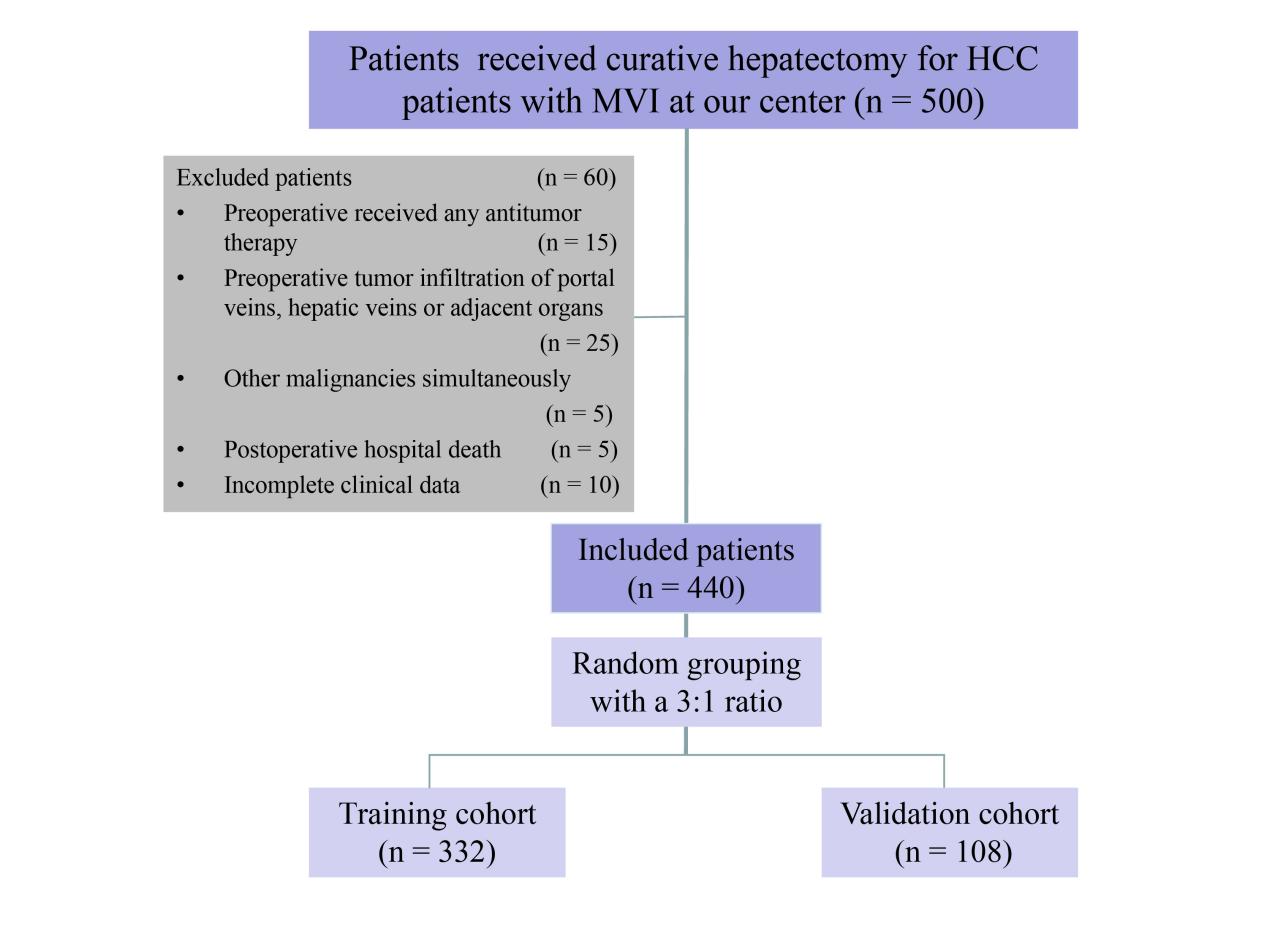
**

**Supplementary Figure 1.** Flowchart illustrating patient selection, exclusion criteria, and cohort allocation used for CART model development and validation.


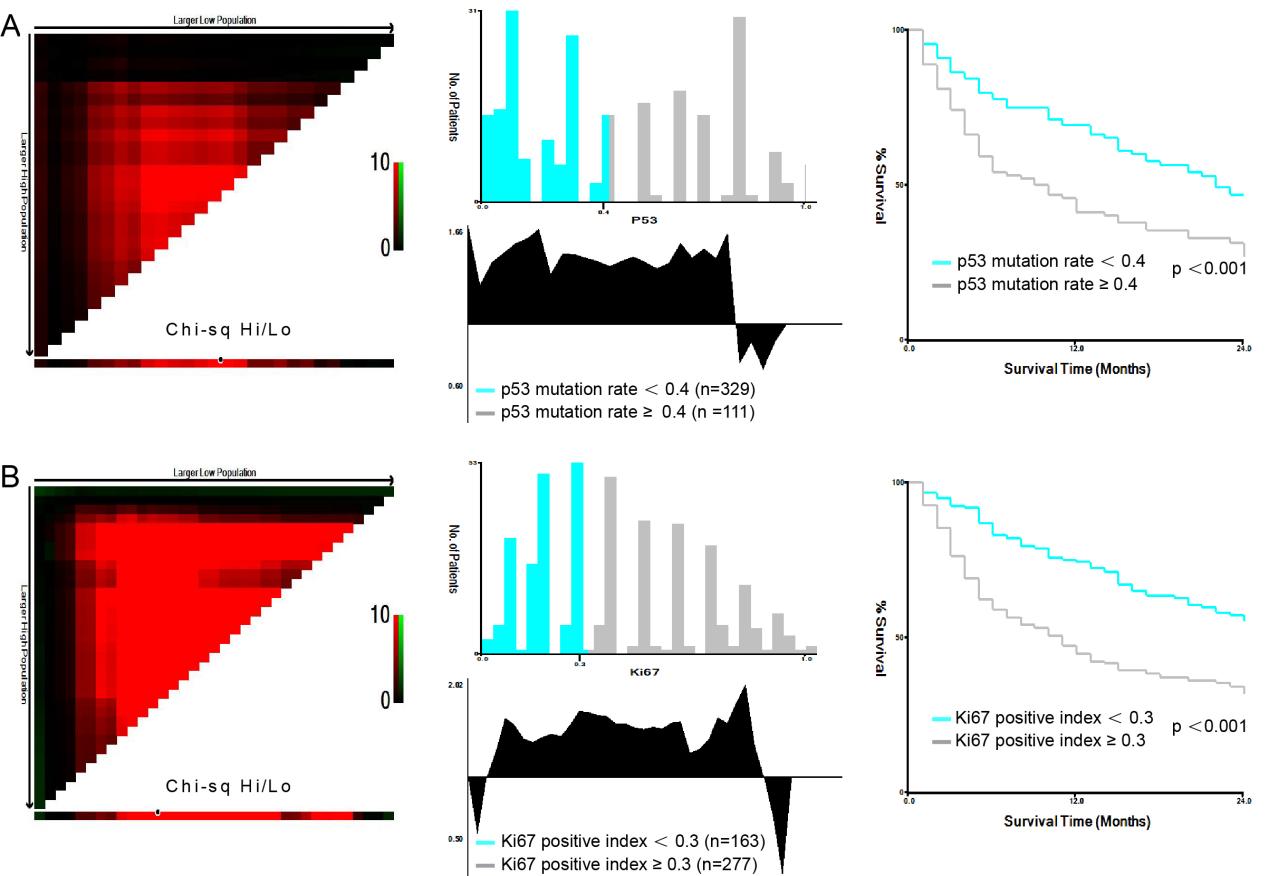


**Supplementary Figure 2.** Optimal cut-off values were defined using X-tile software: 0.4 for the p53 mutation rate (A) and 0.3 for the Ki-67 index (B). These thresholds were applied to stratify patients by ER risk and were subsequently used in CART model training on the training cohort.

*Abbreviations: ER, early recurrence; X-tile, Yale University (version 3.6.1); Ki-67, proliferation marker protein.*


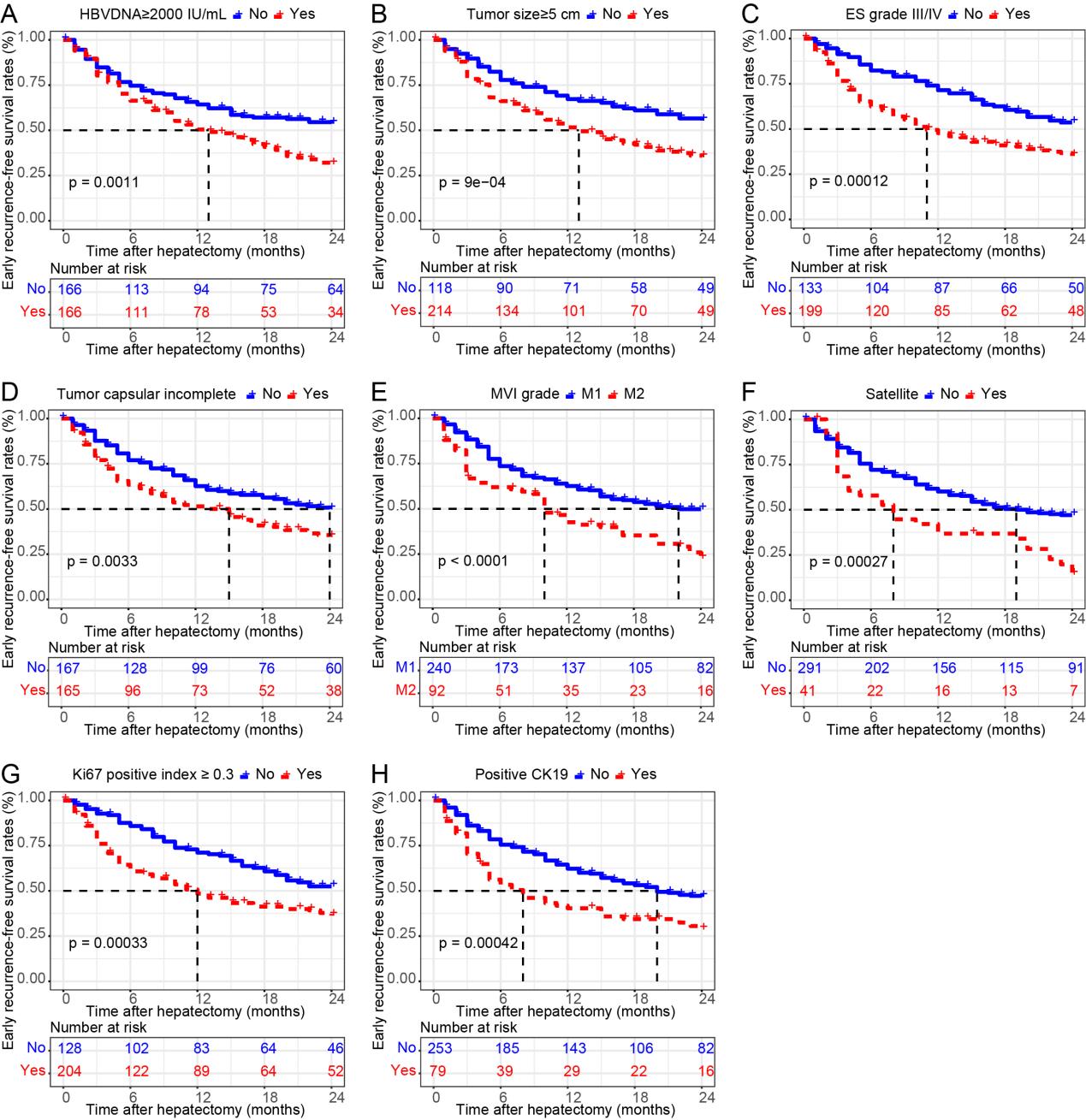


**Supplementary Figure 3.** Recurrence-risk stratification for eight prognostic variables in the training cohort. (A) HBV-DNA; (B) Tumor size; (C) Edmondson-Steiner (ES) grade; (D) Tumor capsule; (E) MVI classification; (F) Satellite nodules; (G) Ki-67 index; (H) CK19 expression.

*Abbreviations: HBV-DNA, hepatitis B virus DNA load; MVI, microvascular invasion; CK19, cytokeratin 19.*


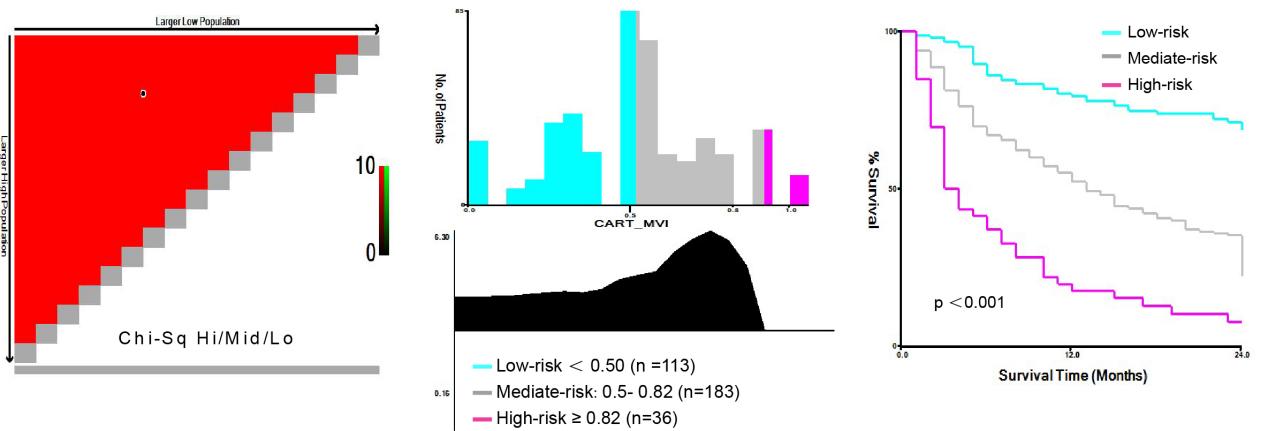


**Supplementary Figure 4.** Risk group classification using the CART model based on cut-off values determined by X-tile software. Risk groups were defined as follows: low (<0.50), intermediate (0.50–0.82), and high (≥0.82) based on CART-predicted probability.

*Abbreviations: ER, early recurrence; CART, classification and regression tree; X-tile, Yale University (version 3.6.1).*
